# Ethnobotanical Survey and Documentation of Medicinal Plants Used to Manage Snakebite Envenomation in Nyatike Subcounty, Migori County, Kenya

**DOI:** 10.1155/tswj/5556367

**Published:** 2025-11-06

**Authors:** Prince Ojuka, George S. Nyamato, Cleydson B. R. Santos, Njogu M. Kimani

**Affiliations:** ^1^Department of Physical Sciences, University of Embu, Embu, Kenya; ^2^Laboratory of Modeling and Computational Chemistry, Department of Biological Sciences and Health, Federal University of Amapá, Macapá, Amapá, Brazil; ^3^Graduate Program in Biotechnology and Biodiversity-Network BIONORTE, Federal University of Amapá, Macapá, Brazil; ^4^Natural Product Chemistry and Computational Drug Discovery Laboratory, University of Embu, Embu, Kenya

**Keywords:** *Bidens pilosa*, envenomation, ethnobotany, ethnomedicine, medicinal plants, snakebite, traditional knowledge, traditional medicine

## Abstract

**Ethnobotanical Relevance:**

Traditional medicine plays a crucial role in managing snakebite envenomation (SBE) among the people of Nyatike subcounty. This region is particularly important for such a study due to its high incidence of snakebite cases, limited access to healthcare facilities, and strong reliance on indigenous knowledge systems. However, the medicinal plants used for this purpose remain largely undocumented, leading to a lack of scientific investigation. The absence of proper documentation poses a risk of losing this valuable knowledge, as it is primarily passed down orally within trusted families. There is a growing concern that this traditional wisdom may be lost over time due to death, aging of knowledge holders, or declining interest among younger generations.

**Aim of the Study:**

This study sought to identify and document the medicinal herbs most commonly used to manage SBE in Nyatike subcounty. By capturing this ethnobotanical knowledge, the study seeks to promote the preservation of folk medicine and provide a foundation for future pharmacological investigations into their potential antivenom properties.

**Methods:**

Ethnobotanical surveys were conducted between December 2024 and January 2025, involving approximately 60 purposefully selected respondents. To document local knowledge on medicinal plants, the study utilized oral interviews, field walks, and semistructured questionnaires.

**Results:**

Thirteen medicinal plant species from seven different families were identified and documented for managing SBE in Nyatike subcounty. The Asteraceae family was the most represented, with four species. Key patterns revealed that the most frequently cited species, *Combretum collinum* Fresen (RFC = 0.75), *Bidens pilosa* L. (RFC = 0.42), *Ozoroa insignis* Delile (RFC = 0.33), and *Tithonia diversifolia* (Hemsl.) A. Gray (RFC = 0.22), are readily accessible, fast-acting, and commonly used by traditional healers to manage SBE.

**Conclusion:**

The study highlights the extensive use of medicinal plants for SBE management in Nyatike subcounty due to the limitations associated with accessing conventional antivenom. These plants may offer promising leads for the development of plant-based antivenoms, either as complementary or as alternative therapies to current treatments. Further research is necessary to isolate the bioactive compounds present in these plants, assess their safety profiles, and evaluate their antivenom potential.

## 1. Introduction

Snakebite envenomation (SBE) is among the leading causes of death globally and is recognized by the WHO as a neglected disease [1]. Annually, over five million snakebites happen worldwide, with 138,000 fatalities and 400,000 resulting in permanent disabilities [1, 2]. In Africa, approximately 500,000 cases are reported annually, including over 70,000 from East Africa, a situation worsened by the change in climate and deforestation, which disrupt snake habitats, forcing them to relocate into populated areas [3–5]. In Kenya, snakebite deaths were recorded as 89, 67, and 22 in 1971, 1972, and 1973, respectively [3], with 151 cases reported in 1994, 19% of which were from venomous snakes [3]. Between 2007 and 2016, 7772 Kenyans were maimed by snakebites, resulting in 614 deaths, and Baringo County alone reported 300–500 cases per month during this period [3]. These incidents are most frequent at the beginning of the rainy season when snakes emerge to hunt and breed [6, 7]. On average, 15–25 Kenyans die each day from SBE, and over a hundred suffer amputations leading to permanent disability [3, 5]. The majority of bites occur when victims accidentally step on snakes while unprotected or barefoot [8], though some are caused by intentional actions or alcohol-induced behavior [9]. Snake venom injected through bites triggers both local and systemic toxic effects, which can have severe consequences [10].

According to the Kenya Wildlife Service, venomous snakebite compensation claims accounted for about 81% of all cases related to human–wildlife conflicts, involving claims totaling millions of Kenyan shillings over the decades. This financial burden led the Ministry of Tourism and Wildlife to recognize the situation as a crisis threatening national bankruptcy, eventually resulting in snakebites being excluded from the catalog of eligible human–wildlife conflict compensation claims [3]. The primary treatment for snake envenomation is antivenom therapy [4, 6, 7], but it is associated with side effects like hypersensitivity reactions, limited effectiveness against local tissue damage, and instability [3]. For example, commonly used antivenoms such as F(ab⁣′)_2_-based antivenoms and whole IgG antivenoms have been associated with early adverse reactions like anaphylaxis, pyrogenic responses, and serum sickness, particularly when derived from equine or ovine sources. In addition, antivenoms are typically species-specific, which poses a challenge in regions where multiple venomous snake species coexist [11, 12]. Additionally, antivenoms must typically be administered within 30 min–6 h of the bite to be effective. Moreover, their efficacy can be compromised by regional variation in venom makeup and antigenic reactivity due to the diverse range of venomous snake species [13]. The expensive nature of antivenoms, their limited availability in healthcare facilities, and difficulties accessing hospitals in remote areas contribute to delays in treatment, resulting in high morbidity and mortality rates [8]. Additionally, like many poverty-related diseases, SBE suffers from a lack of public health policies, strategies, and investment, exacerbated by political apathy and the socioeconomic profile of the impacted population [4, 14, 15]. The global shortage of antivenom sera has prompted efforts to discover alternative snake antivenom from other sources [10, 16], to supplement those derived from horses [17]. As a result, up to 85% of snakebite victims in Kenya turn to folk medicine practitioners or employ rudimentary techniques for venom neutralization [3]. Traditional healers provide the frontline defense against snakebite incidents, with their success being difficult to explain owing to their unverified therapeutic constituents and the mystical aspects of their practices [18]. However, victims' direct accounts attest to the effectiveness of their treatments. Despite being largely disregarded by biomedicine, these practitioners treat more snakebite cases than modern healthcare providers [7, 18]. In Kenya's Nyanza region, particularly Nyatike subcounty in Migori County, there is a notably higher incidence of SBE caused by venomous snakes. This is due to the region's hot, dry climate, which puts residents at greater risk of snakebites [19]. The situation is further exacerbated by the inadequate remedies for SBE in many medical centers, particularly in remote areas, and the high cost of antivenom therapies, which many people cannot afford, making it more challenging to manage snakebites in this region [20]. These make this region important for such a study. Medicinal herbs are essential in maintaining human health, particularly in remote zones with constrained access, where specific antivenoms are limited [21]. In Nyatike subcounty, residents often turn to folk medicine, which includes the application of various plants. Locally referred to as “yedhe thuol” (medicine for snake envenomation), these plants are commonly used in traditional practices for managing snakebites [18].

While these plants help bridge the gap in antivenom unavailability, only a small number have been scientifically studied, despite their longstanding use in traditional medicine [4]. Rigorous scientific evaluation is needed to validate the ethnobotanical beliefs and contribute to the formulation of safe and efficient remedies for SBE [22]. One challenge in evaluating the potency and potential of these plants is the absence of comprehensive ethnomedical documentation to guide scientific studies [4]. The adoption of medicinal plants for managing SBE has been employed for centuries, especially in rural ethnic communities [23]. However, much of this knowledge remains undocumented. It is often passed down orally to trusted family members [24–27], with a high risk of losing this knowledge if it is not properly recorded and/or if family members are not interested [28]. Therefore, ethnobotanical documentation is crucial for preserving this knowledge, supporting conservation efforts, and advancing research. This study is aimed at (i) identifying and documenting the medicinal plant species traditionally used to manage SBE in Nyatike subcounty, Migori County, Kenya; (ii) determining the most frequently used species based on their relative frequency of citation (RFC); and (iii) preserving indigenous knowledge and providing a scientific foundation for future pharmacological investigations into the antivenom potential of these plants.

## 2. Methods

### 2.1. Study Area

**Figure 1:**
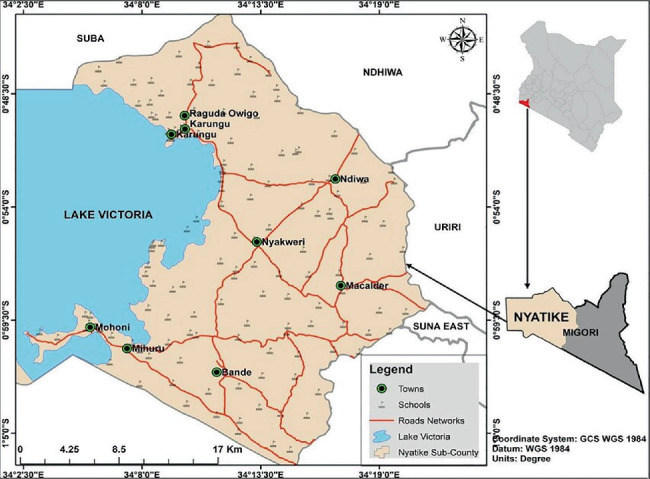
Map of Nyatike subcounty [29].

The plants were collected in Nyatike subcounty, Migori County, Kenya (Figure 1), lying along 34° 10⁣′0″ and 34° 20⁣′0″ E and latitudes 1° 0⁣′0″–0° 10⁣′0″ S [19, 29]. Nyatike subcounty comprises six wards: North Kadem, Muhuru, Kaler, Got Kachola, Macalder/Kanyarwanda, and Kachieng. It is the largest subcounty of Migori County with a population of 176,162 people and has the lowest population density of 260 persons per square kilometer [30]. Nyatike subcounty has an equatorial climate influenced by winds coming from Lake Victoria. Situated at an altitude of 1140 m along the lake's shores, the area experiences yearly rainfall between 700 and 1800 mm, with two rainy seasons. The first season witnesses heavy rainfall, while the second has lighter precipitation [19]. The Intertropical Convergence Zone impacts the seasonal rainfall, and temperatures range from 24°C to 31°C, with high humidity and annual evaporation rates between 1800 and 2000 mm [19]. Nyatike subcounty has an estimated poverty rate of 44%. The area primarily relies on small-scale farming, fishing, mining, and small-scale trading as its main sources of livelihood [29].

### 2.2. Ethnobotanical Data Collection

A field survey was carried out between December 2024 and January 2025. Participants were selected using a purposive sampling method, as outlined by Palinkas et al., targeting 60 individuals (10 from each ward) aged 18–85 years with expertise in medicinal plants used for treating SBE in the study area [31]. The selection criteria included herbalists and knowledgeable community members, individuals familiar with the region, and those who could accurately identify plants by their local names. Initial respondents were identified with assistance from local leaders, residents, and herbalists, who subsequently referred others within their networks. Data collection ceased once no new information emerged regarding medicinal plant use. Ethnobotanical data were gathered through interviews conducted in either Luo or Swahili, based on the participants' preference. Information was also obtained using semistructured questionnaires and guided field visits to plant collection sites. Key details recorded included demographic information (such as name, age, gender, area of expertise, and education level) and botanical aspects, including local plant and snake names, sources, availability, utilized plant parts, preparation and administration methods, potential adverse effects, treatment frequency, and duration. Before participating, all respondents provided informed consent (File S1: The consent form and the questionnaire used to gather ethnomedical data on plants for managing SBE in Nyatike subcounty).

### 2.3. Plant Collection and Identification

Medicinal plants cited by participants as being used for SBE treatment were gathered as voucher specimens. Additionally, photographs of each mentioned plant were taken to aid in identification and documentation (File S2: Photos of medicinal plants captured in situ). A taxonomist identified the collected plant samples, Mr. Patrick Mutiso, at the Faculty of Science and Technology, University of Nairobi. The collected and identified plant specimens were preserved by carefully pressing and drying them before mounting them on archival paper. They were then assigned reference numbers and preserved as voucher specimens.

### 2.4. Data Analysis

Ethnobotanical data were compiled and examined using Microsoft Office Excel 2016, employing descriptive statistical techniques. To determine the most frequently utilized plants for treating SBE in the study area, the RFC method was applied. The RFC for each plant species was calculated by dividing the number of participants who cited a particular plant (frequency of citation, FC) by the total number of respondents (*N* = 60). The RFC index ranged from 0 (indicating that no participants acknowledged the plant's medicinal use) to 1 (signifying that all respondents recognized its usefulness). The calculation followed the formula established by Vitalini et al. [32]:

 $$\begin{array}{c} \text{RFC}=\frac{\text{FC}}{N} \end{array}$$

## 3. Results

### 3.1. Sociodemographic Profile of Study Participants

The research included 60 respondents, ranging in age from 18 to 85 years, who shared ethnobotanical information regarding the medicinal plants utilized for managing SBE in the region. Among the respondents, 60% were male, and 40% were female. The majority (33%) were aged 51–75 years, followed by 30% aged 31–50 years, 22% aged 75 years or older, and 15% aged 18–30. In terms of occupation, only 7% were formally employed, while most participants (55%) relied on farming for their livelihood, while others were small-scale miners and fishermen. Educational levels varied, with 8% having no formal education, 42% completing primary school, 33% attaining secondary education, and 17% holding tertiary qualifications. Most participants (67%) were native to the area, while 8% and 7% were herbalists and traditional healers, respectively. The primary source of ethnomedical knowledge was family and relatives (75%), followed by herbalists (12%) and nontraditional sources such as literature (13%). Regarding ethnomedical experience, 17% of participants had less than 5 years of practice, 28% had 6–10 years, and 55% had over a decade of experience.  Table 1 summarizes the sociodemographic details of the respondents.

### 3.2. Ethnobotanical Data on Documented Plants

**Figure 2:**
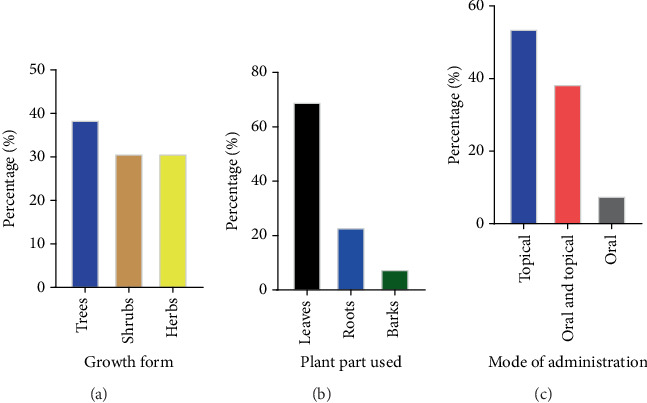
(a) Growth habits of the recorded plants, (b) the plant parts predominantly utilized in remedy preparation for SBE within the study area, and (c) modes of administration of medicinal plants.

Herbal medicines used for managing SBE in Nyatike subcounty were documented along with relevant details, as summarized in Table 2. The study identified 13 medicinal plant species from 7 different families. Among these, the Asteraceae family was the most represented, with four species, then Malvaceae, Anacardiaceae, and Fabaceae with two species each, while families such as Euphorbiaceae, Amaryllidaceae, and Combretaceae were each represented by a single species (Table 2). The species most commonly mentioned included *Combretum collinum* Fresen (RFC = 0.75), *Bidens pilosa* L. (RFC = 0.42), *Ozoroa insignis* Delile (RFC = 0.33), and *Tithonia diversifolia* (Hemsl.) A. Gray (RFC = 0.22), as shown in Table 2. All identified plants were applied to the bite area, with some additionally administered both orally and topically (Table 2). The findings revealed that trees were the most commonly documented plant type (38.5%), followed by herbs and shrubs (30.8% each) (Table 2; Figure 2a). Leaves were the most frequently used plant part in preparing remedies for SBE (69.2%), followed by roots (23.1%), and barks (7.7%) (Table 2; Figure 2b). Modes of administration were categorized as topical, oral, or both. Remedies were predominantly administered topically (53.8%), followed by oral–topical applications (38.5%), and oral (7.7%) (Table 2; Figure 2c).

## 4. Discussion

Herbal medicine holds significant importance in the therapy of various diseases, particularly in rural areas of developing nations [33]. According to a recent WHO report, more than 80% of the world's population depends on herbal plants as their main source of treatment [34]. This growing reliance on herbal remedies can be attributed to their availability, cost-effectiveness, and assumed safety in contrast to modern medicine. Despite their extensive use in traditional practices, only a limited number of medicinal plants have undergone scientific evaluation [35–37]. A major challenge in assessing their effectiveness and advancing their development is the absence of foundational ethnomedical data to support scientific studies. The use of herbal medicine to treat SBE has been a longstanding practice among many rural ethnic communities. Unfortunately, traditional knowledge about such treatments often remains undocumented and is passed orally between trusted family members or relatives [4]. This mode of transmission risks the loss of valuable information, especially when younger generations are disinterested or fail to preserve the knowledge [33]. Documenting ethnomedical practices is therefore crucial for preserving cultural heritage, supporting conservation efforts, and promoting research advancements [7].

### 4.1. Sociodemographic Profile of Study Participants

The research involved 60 respondents aged between 18 and 85 years who provided ethnobotanical information on medicinal plants used in the management of SBE in the region. The sample consisted of 60% males and 40% females, reflecting a slight male dominance in participation, possibly due to gender roles in traditional healing or cultural practices around plant knowledge sharing. The age distribution shows that the majority of participants (33%) were aged 51–75 years, followed by 30% aged 31–50 years, 22% aged 75 years or older, and only 15% aged 18–30 years. This suggests that ethnobotanical knowledge is largely concentrated among older generations, highlighting the risk of knowledge erosion if not documented and passed on effectively [38].

In terms of occupation, only 7% of participants were formally employed, with the majority (55%) depending on farming for their livelihood. Others included small-scale miners and fishermen. This indicates that most participants were part of rural, subsistence-based communities where traditional medicine may serve as a primary form of healthcare due to limited access to conventional health facilities. Educational levels were varied, with 8% having no formal education, 42% having completed primary school, 33% having attained secondary education, and 17% having attained tertiary education. The relatively low level of formal education suggests that traditional knowledge systems are maintained independently of the formal education system, often passed down orally through generations.

Notably, 67% of participants were native to the area, while 8% were herbalists and 7% traditional healers. This highlights the strong community-based foundation of ethnomedical knowledge in the region. The majority of respondents (75%) acquired their knowledge from family and relatives, underscoring the intergenerational transmission of medicinal plant use. Only a small proportion cited herbalists (12%) and literature or other sources (13%) as their sources of information. Regarding experience in the use of ethnomedicine, over half (55%) had more than 10 years of practice, while 28% had 6–10 years and 17% had less than 5 years. This demonstrates that the participants were generally experienced, making their contributions highly valuable for documenting traditional remedies. Collectively, these findings indicate that traditional knowledge related to snakebite treatment is deeply rooted in local culture and predominantly preserved among older, rural community members with limited formal education. The data emphasize the importance of safeguarding this knowledge through scientific validation and integration into broader healthcare frameworks, especially as younger generations may have reduced exposure to these traditions.

### 4.2. Medicinal Plant Knowledge Acquisition

The results of this research reveal that the majority of participants (75%) acquired their ethnomedical knowledge from family members. This aligns with earlier research, such as Uniyal et al., which highlighted the generational verbal transfer of traditional medicinal knowledge [39]. However, younger individuals often view this knowledge as outdated, leading to its potential loss when no formal records are kept.

### 4.3. Growth Habit and Habitats of Medicinal Herbs

In the current research, trees were the most commonly used plant species for managing SBE in the Nyatike subcounty. This may be attributed to their resilience against drought, ensuring year-round availability in the region [40]. Additionally, herbal medicine in the study area was typically found in bushes, gardens, homesteads, and along roadsides, highlighting their abundance and accessibility. Prior studies have demonstrated that the presence of herbaceous vegetation in native ecosystems influences their use for medicinal purposes [39].

### 4.4. Plant Parts Used for Herbal Remedies

The current study further demonstrated that leaves were the most frequently used plant parts in SBE remedies, probably because they are easy to gather and are available in large quantities. Leaves are often preferred in traditional medicine because of their rich phytochemical composition, including bioactive compounds [4]. These phytochemicals are recognized for their healing attributes, contributing to the pharmacological activity of herbal preparations. Interestingly, some participants reported using either single plant parts or combinations of multiple parts to treat SBE. This practice is consistent with earlier research suggesting that combining plant parts can produce synergistic effects [38], enhancing efficacy and accelerating recovery [3].

### 4.5. Methods of Preparing and Administering Remedies

The common preparation methods for herbal remedies comprised poultices, powders, decoctions, infusions, and tinctures, which were applied orally, topically, or via both methods. Water served as the main solvent in most remedy preparations [41, 42]. The mode of treatment was found to vary depending on the type of snake, snake envenomation, such as a direct bite, spit venom, accidental encounter, or a “sent snake to bite” perceived enemies, reflecting the participants' understanding of basic pharmacological principles.

### 4.6. Plants Identified With High RFC

The study used the RFC to identify the most commonly used plants for managing SBE [43]. The RFC index reflects the reliability of the information and highlights plants with a longstanding history of use. Among the documented plants, *Combretum collinum* Fresen, *Bidens pilosa* L., *Ozoroa insignis* Delile, and *Tithonia diversifolia* (Hemsl.) A. Gray had the highest RFC values, indicating their frequent use and importance in the local management of SBE. Notably, the documentation of *Ozoroa insignis* Delile, *Erigeron bonariensis* L., and *Tragia brevipes* Pax in the management of SBE represents a novel contribution to the ethnobotanical literature. To date, no known scientific studies have reported the use of either *Ozoroa insignis* Delile, *Erigeron bonariensis* L., or *Tragia brevipes* Pax for antivenom purposes. This makes their identification and traditional use in Nyatike subcounty a significant finding, providing new insights into unexplored therapeutic potentials. In addition, this study is the first to comprehensively document the ethnomedical practices related to snakebite treatment in Nyatike subcounty, a region characterized by frequent snake–human conflict and limited access to formal healthcare services.

### 4.7. Most Used Plant Families

Many families identified in this study are acknowledged for their effectiveness in managing or preventing snakebites in various countries. In Pakistan, Uganda, Tanzania, Djibouti, Ethiopia, and Nigeria, species from the Asteraceae, Fabaceae, and Malvaceae families have been documented for their medicinal use [6, 44–46] . Similarly, in Bangladesh [47, 48], Central America [49], and India [50], the Asteraceae, Euphorbiaceae, Fabaceae, and Combretaceae families have been reported for their role in snakebite management. Moreover, it was observed that only a limited number of botanical species are used for snakebite treatment across multiple locations. This may be due to the widespread presence of similar active compounds in various plant species, particularly within the Asteraceae and Fabaceae families. Additionally, some of the identified plants are also utilized for repelling, deterring, or eliminating snakes to protect both humans and livestock. Almost all the plants highlighted in this study are used to treat various ailments, both in Kenya and other countries. For instance, *Bidens pilosa* L., whether as a whole plant or in parts, has been documented for its medicinal value in managing over 40 conditions, including inflammation, immune disorders, digestive issues, infections, cancer, metabolic syndrome, and wounds [3, 51]. Given their widespread medicinal applications, these plants are often utilized in various communities for managing snakebites, providing a basis for their potential effectiveness.

### 4.8. Snakes: Victim and Witness Experiences

All respondents reported direct or indirect experience with snakebites. Specifically, 100% of participants had either been bitten by a snake themselves or had encountered someone who had been bitten. The most commonly reported snakes included puff adder (*Bitis arietans*) locally known as “fuu,” black-necked cobra (*Naja nigricollis*) “rachier,” and black mamba (*Dendroaspis polylepis*) “olueru.” When asked about the treatment methods used, all respondents indicated the use of plant-based remedies, either alone or in combination with hospital treatment. Sixty percent of respondents exclusively relied on traditional plant remedies, while 40% combined plant-based treatment with hospital interventions. Notably, none of the respondents relied solely on hospital treatment, highlighting a strong cultural reliance on ethnomedicine in snakebite management and a shortage of antisnake venom in the health facilities.

### 4.9. Dosage and Treatment Duration

Regarding dosage and treatment duration, respondents provided detailed information on traditional protocols. The most common dosage form was a decoction, with administration continuing for an average of 5–7 days until symptoms subsided. The exact dosage was determined based on the severity of envenomation and the patient's response to treatment. All respondents confirmed that verbal instructions were given when administering the remedy, ensuring that the correct preparation and application methods were followed. This practice plays a crucial role in knowledge transfer across generations, preserving traditional healing practices. Study participants also demonstrated awareness of the dangers of overdosing and mentioned using substances such as raw egg, cow's milk, and water as antidotes [52, 53]. However, herbalists were often reluctant to disclose the specific adjuvants in their remedies, citing the need for secrecy to preserve the potency of their formulations. This secrecy often involved rituals or offerings, which served as a safeguard against sharing their knowledge openly, which, as a result, limits the sharing of information, particularly in this study [54, 55].

### 4.10. Safety of the Herbal Medicine

When asked about the safety of these plant-based treatments, 100% of participants affirmed their effectiveness and safety in managing snakebites. No adverse effects were reported, and many participants emphasized that these remedies had been used successfully for generations.

Numerous antivenom plants used by herbalists in Kenya remain largely undocumented, despite the significant threat of their extinction due to deforestation, overharvesting, and the loss of traditional knowledge as healers pass away [18, 51]. This challenge is further compounded by the fact that folklore medicine is often kept as a closely held ancestral knowledge and family secret, and as seen in other sub-Saharan countries [18, 56], younger generations are not always inclined to inherit this knowledge, as they spend much of their youth in formal education [4, 18, 57]. Although the majority of antivenom plant species are native and thrive in the wild, certain species, like *Senna siamea* (Lam.) H. S. Irwin & Barneby and *Tithonia diversifolia* (Hemsl.) A. Gray, are naturalized exotics.

## 5. Novelty

To determine the novelty of the documented plant species, the collected ethnobotanical data were cross-referenced with existing literature available in online databases such as Scopus, Web of Science, PubMed, and Google Scholar. This comparison revealed several plant species not previously reported for snakebite treatment, including *Tragia brevipes* Pax, *Ozoroa insignis* Delile, and *Erigeron bonariensis* L. Additionally, variations were observed in the methods of administration and plant parts used compared to existing reports. Notably, many of the recorded species remain underexplored in terms of pharmacological and phytochemical evaluation. Therefore, these plants warrant further phytochemical and pharmacological studies to validate and support their traditional medicinal uses.

## 6. Conclusions and Recommendations

The diverse plant species used in Kenyan communities hold significant potential for treating snake envenomation. The study area demonstrated a rich diversity of medicinal plants and associated indigenous knowledge. Participants possessed varying levels of understanding regarding the use of medicinal plants, which appeared to be influenced by factors such as gender, age, and education level. Generally, men exhibited more ethnobotanical knowledge than women, older individuals were more knowledgeable than the youth, and illiterate participants had a deeper understanding compared to their literate counterparts. The study identified 13 herbal plants used for managing SBE in Nyatike subcounty, Migori County, Kenya, with *Combretum collinum* Fresen, *Bidens pilosa* L., *Ozoroa insignis* Delile, and *Tithonia diversifolia* (Hemsl.) A. Gray being the most frequently cited. Most of these plants are harvested from the wild, prepared as infusions, and administered orally, topically, or through both methods, reflecting the flexibility and depth of traditional healing practices.

However, this traditional knowledge is increasingly endangered due to generational gaps and a lack of systematic documentation. There is a pressing need for conservation strategies to protect both the plant species and the ethnobotanical knowledge associated with them. Preserving this information is crucial for sustaining cultural identity and local health systems. Further research is warranted to validate these traditional remedies through rigorous phytochemical and pharmacological studies. It is recommended that active compounds with a high RFC be isolated from plants and their antivenom potential be assessed using in vitro and in vivo models. Additionally, safety profiles and toxicity studies should be conducted to support potential drug development. These findings can inform national health policies and promote the integration of scientifically validated traditional remedies into official treatment frameworks.

In conclusion, the high RFC values observed underscore the pharmacological promise of these medicinal plants. Their potential contributions to antivenom therapy, combined with the urgency of conserving both flora and knowledge systems, highlight the importance of immediate and multidisciplinary efforts in research, conservation, and policy.

## Figures and Tables

**Table 1:** Sociodemographic data for respondents.

**Parameter**	**Group**	**Number of participants**	**% of participants**
Sex	Male	36	60
	Female	24	40

Age (years)	18–30	9	15
	31–50	18	30
	51–75	20	33
	75 and above	13	22

Education	Primary	25	42
	Secondary	20	33
	Tertiary	10	17
	None	5	8

Type of practice	Herbalist	5	8
	Traditional healer	7	12
	Local community member	40	67
	Others	8	13

Source of income	Employment	4	7
	Business	10	17
	Farming	33	55
	Mining	5	8
	Fishing	5	8
	Others	3	5

Experience (years)	0–5	10	17
	6–10	17	28
	Above 10	33	55

Source of knowledge	Relatives	45	75
	Herbalists	7	12
	Others	8	13

**Table 2:** Plant-based therapies for managing SBE in the study area.

**Plant species (V/N)**	**Luo name**	**Family**	**Growth habit**	**Part(s) utilized**	**Method of preparation**	**Mode of administration**	**FC**	**RFC**
*Tragia brevipes* Pax OP UON 2025/001	Aila matindo	Euphorbiaceae	Herb	Leaves and Roots	The leaves are chewed and applied to the bite site, while the stem or roots are sliced, sun-dried, ground into a powder, and then applied	Topical	4	0.07
*Ammocharis tinneana* (Kotschy & Peyr.) Milne-Redh. & Schweick OP UON 2025/002	Thuon pap	Amaryllidaceae	Herb	Roots	The sap from the roots is utilized to formulate a remedy for snakebite antidotes	Topical oral	3	0.05
*Linzia glabra* Steetz OP UON 2025/003	Olusia	Asteraceae	Shrub	Leaves	The ash from the leaves or crushed leaves is applied to scarifications around the snakebite site as a remedy	Topical	2	0.03
*Bidens pilosa* L. OP UON 2025/004	Anyiego	Asteraceae	Herb	Leaves	The crushed leaves of this plant are applied to fresh wounds as an astringent and a remedy for snakebites	Topical	25	0.42
*Grewia trichocarpa* Hochst. ex A. Rich. OP UON 2025/005	Powo	Malvaceae	Shrub	Leaves	The leaves are crushed and applied to cuts made on the bitten area. Sometimes used with *Combretum collinum* Fresen	Topical	7	0.12
*Combretum collinum* Fresen OP UON 2025/006	Adugo	Combretaceae	Tree	Roots	The roots are used to prepare a snakebite antidote, typically administered through scarification. Sometimes used with *Grewia fallax* K	Oral topical	45	0.75
*Searsia natalensis* (Bernh. ex C. Krauss) F.A. Barkley OP UON 2025/007	Sangla	Anacardiaceae	Tree	Leaves	The roots and leaves are utilized to create an antidote for snakebites, usually applied through scarification. It is sometimes combined with *Grewia fallax* K, *Ozoroa insignis* Delile, and *Combretum collinum* Fresen	Oral topical	6	0.1
*Tithonia diversifolia* (Hemsl.) A. Gray OP UON 2025/008	Akech	Asteraceae	Shrub	Leaves	A leaf infusion is given orally as a remedy for snakebite	Oral	13	0.22
*Ozoroa insignis* Delile OP UON 2025/009	Mathare	Anacardiaceae	Tree	Leaves	A leaf infusion is given orally as a remedy for snakebite	Oral topical	20	0.33
*Erigeron bonariensis* L. OP UON 2025/010	No luo name	Asteraceae	Shrub	Leaves	An infusion of the leaves is taken orally as a treatment for snakebites	Topical	5	0.08
*Erythrina excelsa* Baker OP UON 2025/011	Roko	Fabaceae	Tree	Barks	The sap from the bark serves as a remedy for snakebites	Topical	8	0.13
*Senna siamea* (Lam.) H. S. Irwin & Barneby OP UON 2025/012	Ndek owino	Fabaceae	Tree	Roots	The roots of this tree are utilized as a snakebite antidote	Oral topical	1	0.02
*Corchorus trilocularis* L. OP UON 2025/013	Apoth	Malvaceae	Herb	Leaves	An infusion is applied by dropping or sprinkling it into the eye to counteract snake venom that has been spat into the eye	Topical	7	0.12

Abbreviations: FC, frequency of citation; RFC, relative frequency of citation; V/N, voucher number.

## Data Availability

The data supporting this research's findings are contained within the article and in the supporting information. The authors can provide any further data upon reasonable request.

## Supporting Information

Supporting Information Additional supporting information can be found online in the Supporting Information section. The consent form, the questionnaire used to gather ethnomedical data on plants for managing SBE in Nyatike subcounty, and photos of the collected medicinal plants captured in situ are available in the supporting file.
