# Supplementary material for: Ethnobotanical Survey and Documentation of Medicinal Plants Used to Manage Snakebite Envenomation in Nyatike Subcounty, Migori County, Kenya
Source: ScientificWorldJournal. 2025 Nov 6;2025:5556367. doi: 10.1155/tswj/5556367 (PMC12615033; doi:10.1155/tswj/5556367)
Supplement: Supporting Information — Additional supporting information can be found online in the Supporting Information section. The consent form, the questionnaire used to gather ethnomedical data on plants for managing SBE in Nyatike subcounty, and photos of the collected medicinal plants captured in situ are available in the supporting file. [file 5556367.f1.docx]

**Supplementary file 1:** **The consent form, the questionnaire used to gather ethnomedical data on plants for managing SBE in Nyatike Sub-County**

**Consent Form**

**Title of Study**: Ethnobotanical Survey of Medicinal Plants Used for Snakebite Envenomation in Nyatike subcounty, Kenya.

**Principal Investigator**: Prince Ojuka
**Study Location**: Nyatike Sub County, Migori County, Kenya
**Purpose of Study**: Academic (PhD. Research)

Dear Participant,

You are invited to participate in this study conducted by Prince Ojuka, a postgraduate student pursuing PhD in Chemistry at the University of Embu.

The purpose of this study is to gather information about medicinal plants used to manage snakebite envenomation in this area, focusing on the parts of the plants used, methods of preparation, and routes of administration and the types of snakes common in this area. A structured questionnaire will be used to collect this data.

**Confidentiality:**
All information you provide will be treated as confidential and used solely for research purposes. Participation is entirely voluntary, and you may withdraw at any time without any consequences, even if you initially agree to participate.

**Potential Benefits:**

This study aims to document ethnomedical knowledge for heritage preservation and to advance scientific understanding of snakebite management.

**Potential Risks:**
There are no known risks associated with participating in this study.

**Participant Declaration:**
I confirm that I am a native resident or herbalist in this region. I understand the purpose of the study and feel capable of answering questions regarding traditional medicines used for snakebite envenomation.

Name:……………………………………………..
Signature:………………………………………….
Contact Information:……………………………….

Note:
If you have any questions about this study, please feel free to ask now or contact me at the following address:
P.O. BOX 6-60100, Embu
Phone Number: 0706711334

**ETHNOBOTANICAL DATA COLLECTION QUESTIONNAIRE**

**Instructions:**
Please fill the blank spaces and select the appropriate option(s) by ticking the box(es) provided.

|  |
| --- |

**Respondent's Personal Information**

1. **Gender:**
   ☐ Male
   ☐ Female
2. **Age Category:**
   ☐ 18–30 years
   ☐ 31–50 years
   ☐ 51–75 years
   ☐ 75 and above
3. **Level of Education:**
   ☐ Primary
   ☐ Secondary
   ☐ Tertiary
   ☐ Others, specify: ………………………………………….
4. **Primary Source of Income:**
   ☐ Employment
   ☐ Business

☐ Farming

☐ Fishing

☐ Mining
☐ Others, specify: ………………………………………….

|  |
| --- |

**Experience in Managing Venomous Bites**

1. **Type of Practice:**
   ☐ Herbalist
   ☐ Traditional Healer
   ☐ Local Community Member
   ☐ Others, specify: ………………………………………….
2. **Years of Experience in This Field:**
   ……………………………………
3. **How Did You Acquire Knowledge?**
   …………………………………………………………
4. **Are you familiar with the occurrence of snakebites in this Area?**
   ☐ Yes
   ☐ No
5. **What are the common snakes found in this area?**
6. ……………………………………………………………………………………
7. ……………………………………………………………………………………
8. ……………………………………………………………………………………
9. ……………………………………………………………………………………
10. ……………………………………………………………………………………
11. **Have you been bitten by a snake?**
    ☐ Yes
    ☐ No
    If yes, which snake bit you?
    ……………………………………………………………………………………

what plant(s) or combinations do you use in managing the bite?
……………………………………………………………………………………………
……………………………………………………………………………………………
……………………………………………………………………………………………

1. **Did you recover using plants, or did you visit a hospital?**
   ☐ Treated with plants
   ☐ Hospital treatment
   ☐ Both
2. **Have you encountered someone bitten by a snake?**☐ Yes
   ☐ No

If yes, which snake bit him/her?
……………………………………………………………………………………

what plant(s) or combinations used in managing the bite?
……………………………………………………………………………………………
……………………………………………………………………………………………

1. **Where do you source the plant(s) used in treatment?**
   ……………………………………………………………………………………………
2. **Are these plants readily accessible?**
   ☐ Readily available
   ☐ Scarce
   **Seasonally Available (specify the season):**

☐ Cold
☐ Hot

|  |
| --- |

**Preparation and Use of Plants**

1. **Which part(s) of the plant do you use for preparing the treatment?**
   ☐ Leaves
   ☐ Bark
   ☐ Roots
   ☐ Flowers
   ☐ Pulp
   ☐ Fruit
   ☐ Seed
2. **How is the plant prepared for use?**
   ☐ Dried
   ☐ Fresh
   ☐ Others, specify: ………………………………………….
   ……………………………………………………………………………………………
3. **How is the prepared remedy administered?**
   ☐ Oral
   ☐ Topical application
   ☐ Others, specify: ………………………………………….
4. **What is the recommended dosage or quantity used?**
   ………………………………………………………………
5. **For how long is the remedy administered?**
   ………………………………………………………………
6. **Do you give verbal instructions when administering this remedy?**
   ☐ Yes
   ☐ No
7. **Is the use of these plants safe for treating snakebites?**☐ Yes
   ☐ No

|  |
| --- |

**Thank you for your participation**

**Supplementary file 2: Photos of medicinal plants captured in situ**


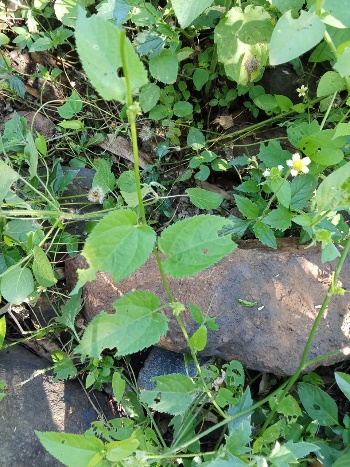

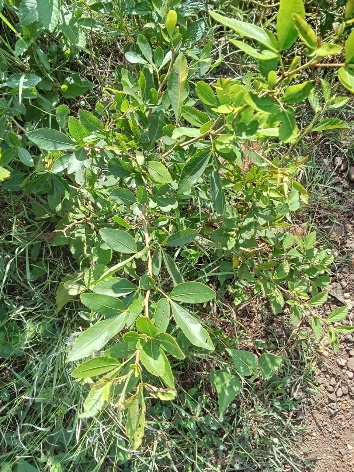

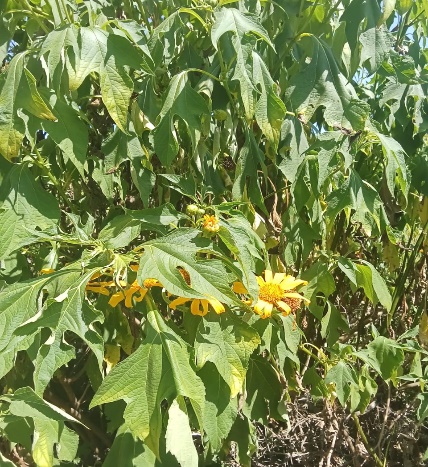


OP UON 2025/001 OP UON 2025/007 OP UON 2025/008


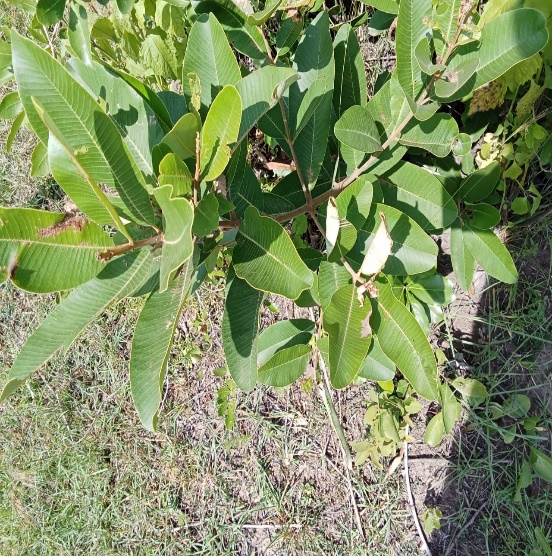

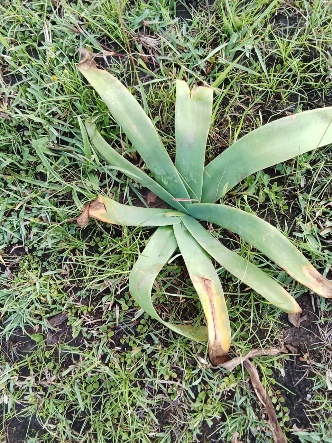

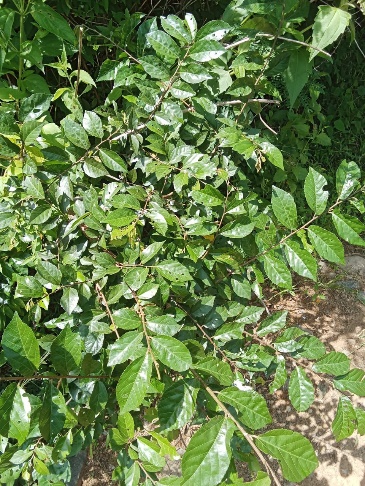


OP UON 2025/009 OP UON 2025/002 OP UON 2025/005


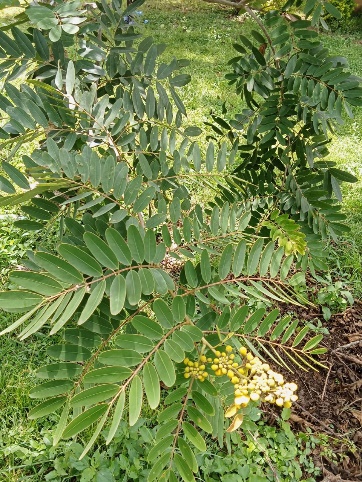

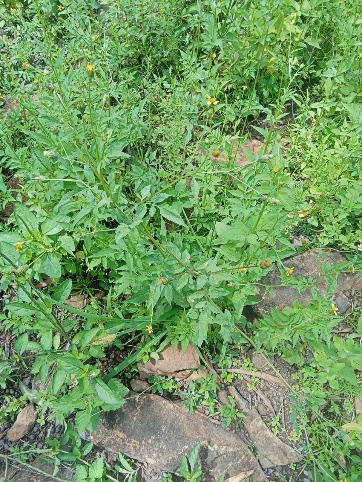

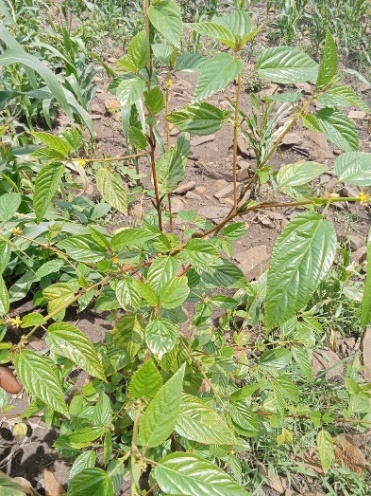


OP UON 2025/012 OP UON 2025/004 OP UON 2025/013


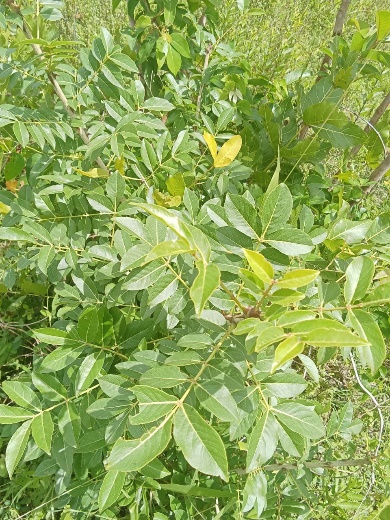

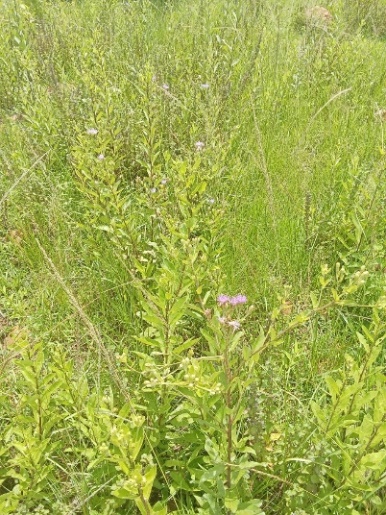

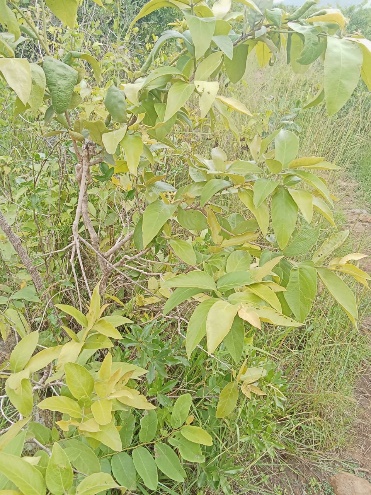


OP UON 2025/011 OP UON 2025/003 OP UON 2025/006


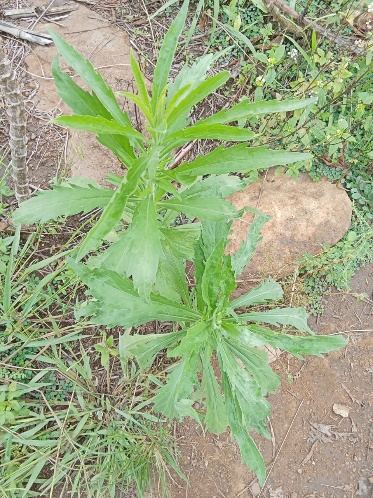


OP UON 2025/010
